# Focal Adhesion Kinase Signaling Mediated the Enhancement of Osteogenesis of Human Mesenchymal Stem Cells Induced by Extracorporeal Shockwave

**DOI:** 10.1038/srep20875

**Published:** 2016-02-11

**Authors:** Jun Hu, Haojie Liao, Zebin Ma, Hongjiang Chen, Zhonglian Huang, Yuantao Zhang, Menglei Yu, Youbin Chen, Jiankun Xu

**Affiliations:** 1 Department of Orthopaedics, the First Affiliated Hospital, Shantou University Medical College, Guangdong Province, China; 2 Department of Orthopaedics and Traumatology, Prince of Wales Hospital, Faculty of Medicine, the Chinese University of Hong Kong, Hong Kong SAR, China; 3 The Sun-Yat-Sen Memorial Hospital, Sun-Yat-Sen University, Guangdong Province, China

## Abstract

Extracorporeal shockwave (ESW) has been shown of great potential in promoting the osteogenesis of bone marrow mesenchymal stem cells (BMSCs), but it is unknown whether this osteogenic promotion effect can also be achieved in other MSCs (i.e., tendon-derived stem cells (TDSCs) and adipose-derived stem cells (ADSCs)). In the current study, we aimed not only to compare the osteogenic effects of BMSCs induced by ESW to those of TDSCs and ADSCs; but also to investigate the underlying mechanisms. We show here that ESW (0.16 mj/mm^2^) significantly promoted the osteogenic differentiation in all the tested types of MSCs, accompanied with the downregulation of miR-138, but the activation of FAK, ERK1/2, and RUNX2. The enhancement of osteogenesis in these MSCs was consistently abolished when the cells were pretreated with one of the following conditions: overexpression of miR-138, *FAK* knockdown using specific siRNA, and U0126, implying that all of these elements are indispensable for mediating the effect of ESW. Moreover, our study provides converging genetic and molecular evidence that the miR-138-FAK-ERK1/2-RUNX2 machinery can be generally activated in ESW-preconditioned MSCs, suggesting that ESW may be a promising therapeutic strategy for the enhancement of osteogenesis of MSCs, regardless of their origins.

Extracorporeal shockwave (ESW) has been widely used in musculoskeletal disorders such as bone defects (delayed- and non-union of bone fractures, avascular necrosis of femoral head), with a success rate around 80%, while the complications are low or negligible^1^. ESW in orthopaedics is not used to disintegrate tissues, rather to induce tissue repair and regeneration^2^. Several ESW devices have been approved by the United States Food and Drug Administration (FDA) as Class III orthopedic lithotripsy devices^3^.

ESW causes both direct (physical) and indirect (biological) effects on the interfaces of treated tissue. Regarding the physical effects, both the positive and the negative phase of ESW have effects on tissues^4^. However, it seems that the biological effects of ESW, which transduce the mechanical signals into biological signals, is more crucial for the tissue repair. Mechanotransduction stimulates extracellular matrix binding proteins and the nucleus via the cytoskeleton resulting in response leading to tissue regeneration. Several previous studies have demonstrated that ESW promotes bone healing through a typical biological response characterized by the up-regulation of bone growth factors, bone morphogenetic proteins^5^,^6^,^7^, and the activities of extracellular signal-regulated kinases (ERKs) and p38 kinase^8^. It has also been reported that an ESW treatment at 0.16 mJ/mm^2^ for 500 impulses induces osteogenesis *in vitro* partially through a rapid membrane hyperpolarization and activation of Ras^9^. The trabecular bone volume fraction and trabecular thickness of the treated calcaneus were significantly enhanced by ESW in an ovariectomized goat model^10^. Moreover, clinical patients with fracture nonunion have been also benefiting from ESW treatment^11^,^12^. However, previous *in vitro* studies mainly focused on the bone marrow mesenchymal stem cells (BMSCs)^13^,^14^ or osteoblasts^15^, while *in vivo* studies were just observational without providing detail mechanisms. It is unknown whether the osteogenic stimulation effect could also be achieved in other types of MSCs, i.e. adipose-derived stem cells (ADSCs)^16^,^17^,^18^ and tendon-derived stem cells (TDSCs)^19^,^20^,^21^; and whether there is a common mechanistic foundation by which ESW promotes the osteogenesis in the mentioned types of stem cells. Thus, the cellular and molecular mechanisms underlying the osteogenic effect induced by ESW remain poorly defined.

Focal adhesion kinase (FAK) is involved in the osteogenic generation of new bone^22^,^23^. FAK deficiency in osteoblasts and osteocytes results in delayed bone healing and remodeling *in vivo* and interrupts the response of bone marrow cells to anabolic mechanical stimuli in a tibial injury model^22^,^23^. In addition to earlier studies that highlighting FAK is a crucial player involved in mechanotransduction^22^,^24^, our previous investigation is also able to provide the direct evidence suggesting the link between FAK and ESW^15^. Phosphorylated FAK at the Tyr397 site is a critical factor for mediating the ESW induced adhesion and migration of osteoblasts^15^. Taken together, FAK signaling pathway plays multiple roles in the bone tissue regeneration.

Recently, several studies yielded consistent results that TDSCs and ADSCs were mechano-sensitive to tensile loading^20^,^25^ or fluid shear stress^26^ by differentiating into osteoblasts, implicating they might have similar performance with BMSCs when suffering ESW treatment. Thus, we hypothesized that ESW (0.16 mJ/mm^2^, 500 impulses) may exerts a positive effect on the osteogenic differentiation of MSCs, regardless of their origins, in which the FAK signaling, together with the upstream and downstream factors is indispensable.

On the basis of a series of genetic experiments, our present findings represent the first direct evidence, implicating ESW (0.16 mJ/mm^2^) in the cell fate control of MSCs by intensely promoting their differentiation into osteoblastic lineage, and place the shared “miR-138-FAK-ERK1/2-RUNX2” pathway as the nexus of mechanisms that regulate the cellular response to ESW.

## Results

**Figure 1:**
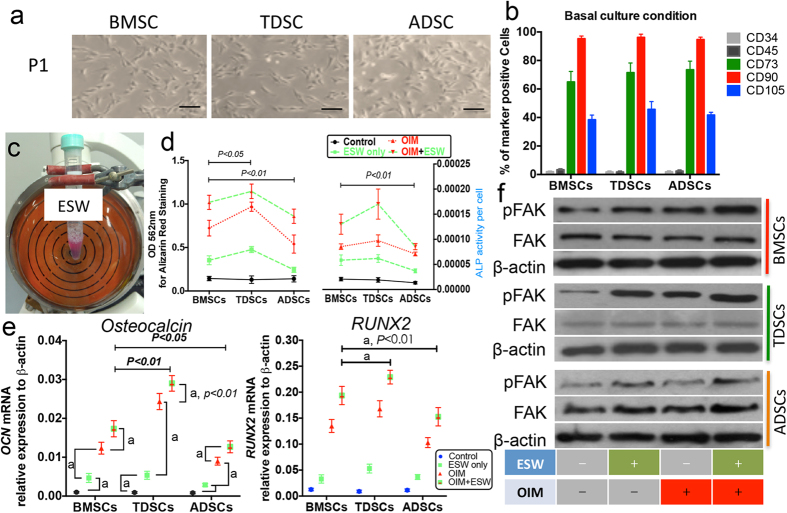
ESW promotes the osteogenic differentiation of MSCs. (**a**) Micrographs showed the similar morphology of the cultured human BMSCs, TDSCs and ADSCs. (**b**) The isolated cells were presented with similar positive expression of CD73, CD90, and CD105. (**c**) Cell suspensions (1.0 × 10^6^ cells in 2 ml complete culture medium) were subjected to ESW treatment. (**d**) Both alizarin Red staining and ALP activity shows that single ESW treatment can promote the osteogenic differentiation in all the tested three types of MSCs. (**e**) The transcriptional expression levels of *osteocalcin* and *RUNX2* were determined using quantitative real-time PCR. Both *osteocalcin* and *RUNX2* mRNA were significantly elevated in ESW groups, at 2 weeks post treatment. (**f**) The expressions of FAK and its phosphorylated form at the site of tyrosine 397 (p-FAK) were analyzed by western blotting. Representative images demonstrated that the FAK signaling pathway was activated within the first 4 hours post-ESW treatment in the tested MSCs. *a, P < 0.01* as compared to the indicated group, using ANOVA with Bonferroni *post hoc* test. Results are presented with mean ± S.D. (error bars) calculated from four paired triplicate experiments. Scale bars, 100 μm.

First, we isolated the primary BMSCs, TDSCs and ADSCs from human tissues (Fig. 1a), and then confirmed all three types of cultured MSCs showed similar percentages of cells with a positive signal of CD34, CD45, CD73, CD90 and CD105, respectively (Fig. 1b). The results are meaningful for addressing the concern that whether the different response to ESW, if any, is owing to distinct expression of stem cell markers, as it has been suggested that certain subpopulation of MSCs with different expression levels of surface markers exerted imbalanced differentiation^21^. Finally, we examined the effect of ESW (0.16 mJ/mm^2^, 500 impulses) on the osteogenic differentiation of MSCs (Fig. 1c).

### ESW promotes the osteogenic differentiation of MSCs via activating FAK

In the current study, it is clearly demonstrated that ESW can enhance the osteogenic differentiation of all tested MSCs, which is evidenced by increased calcium mineral deposition and APL activity, at day 21, with absence or presence of extra osteogenic reagents (Fig. 1d). However, the sensitivity of MSCs to ESW is following the sequence as: TDSCs > BMSCs > ADSCs (Fig. 1d), which is consistent with the previous studies that showing the different osteogenic potential they originally kept, though all of them were classified as MSCs^27^,^28^.

To further confirm the osteogenic ability of ESW, the transcriptional profiles of two known osteoblast markers, *osteocalcin* and *RUNX2*, in hMSCs cultured with different conditions were identified by real-time PCR. At day 14, ESW alone significantly increased the mRNA expression of *osteocalcin* and *RUNX2* as compared to control group cultured in basal medium (*P < 0.01*, Fig. 1e). Synergistic effect was observed when cultured the MSCs in OIM plus ESW, as compared to OIM group (*P < 0.01*, Fig. 1e). The different extents of the ESW enhanced the mRNA expression of *osteocalcin* and *RUNX2* in the tested MSCs were consistent with the results from Alizarin red staining and ALP activity (Fig. 1d,e).

**Figure 2:**
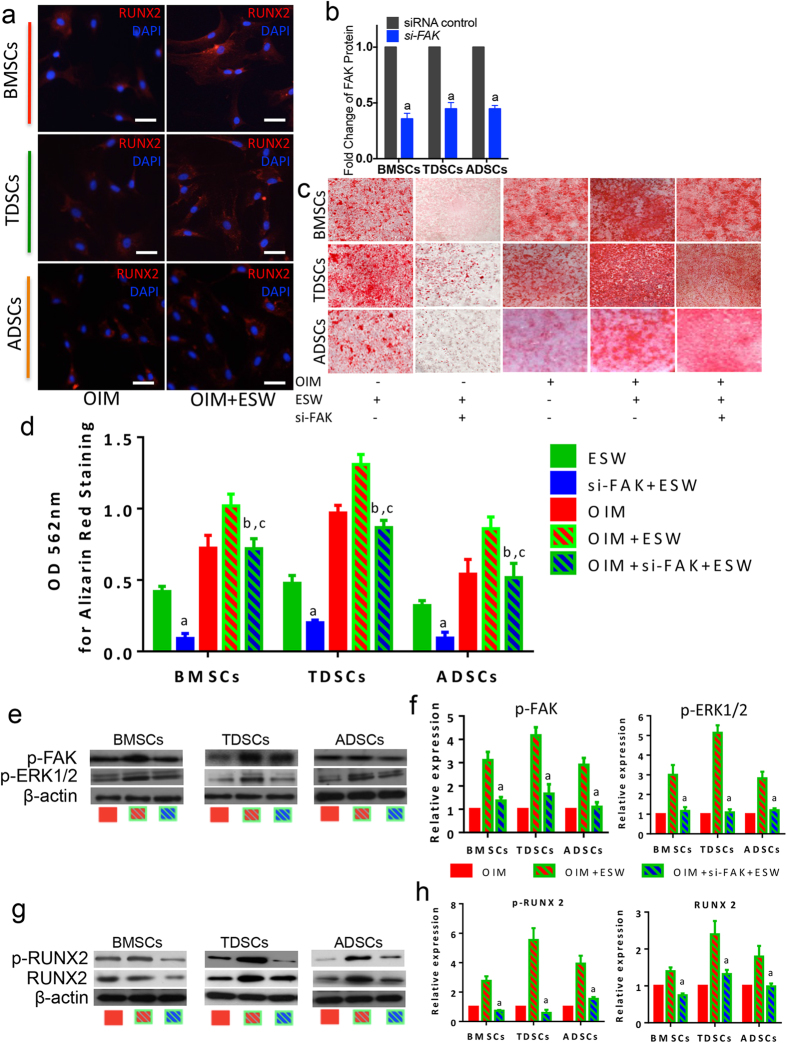
The activation of FAK signaling is common but crucial for the promotion of osteogenesis stimulated by ESW. (**a**) Immunofluorescence assays show the expression of RUNX2 (in Red color) after ESW treatment under the culture environment with osteogenic induction medium (OIM). Scale bars, 50 μm. (**b**) The tested three types of MSCs were successfully transfected with *FAK* siRNA. (**c**) Results from Alizarin red staining show the enhanced capability of calcium nodule formation was significantly blocked in the group pretreated with *FAK* siRNA. (**d**) Quantitative data from (**c**). *a, P < 0.01* as compared to ESW group from the same cell type; *b, P > 0.05* as compared to OIM group from the same cell type; *c, P < 0.01* as compared to OIM plus ESW group from the same cell type, using ANOVA with Bonferroni *post hoc* test. (**e**–**h**) Representative images from western blotting together with the quantitative results show that both the activation of ERK1/2 and RUNX2, the proposed response to ESW, were markedly inhibited in FAK siRNA group. *a, P < 0.01* as compared to OIM plus ESW group. Error bars, mean ± S.D. All figure panels: representative data from at least three independent experiments using biological replicates (n = 4).

On the top of the scientific findings that we have previously revealed the ESW with the same intensity could significantly promote the cell adhesion via activating FAK at the site of tyrosine 397 in rat osteoblasts and FAK signaling has been identified as an essential element during the osteogenic differentiation of hBMSCs^15^,^22^,^29^, we sought to examine whether FAK signaling is also generally involved in the promotion/induction of osteogenesis in ESW treated MSCs, regardless of their origins. As expected, in the tested three types of MSCs, p-FAK was significantly increased after ESW treatment for 4 hours, with or without OIM, whereas the total FAK was not affected (Fig. 1f). Moreover, the protein expression of RUNX2 was also steadily increased in ESW treated MSCs, on day 14, as observed in immunofluorescence assays (Fig. 2a).

### FAK activation is necessary for the promotion of osteogenesis induced by ESW

To identify whether the activation of FAK is required for the stimulation of osteogenic differentiation in human MSCs, we generated MSCs with impaired *FAK* using genetic modification, i.e. specific siRNA targeted for *FAK* gene (supplementary Fig. 1a). After confirming the cells have been successfully transfected, which presented with significant FAK downregulation (Fig. 2b), while without leading to significant cytotoxity (data not shown), the cells were then subjected to ESW treatment together with OIM. As shown in Fig. 2c, accumulated mineralized matrix deposition was visualized by Alizarin red staining in ESW treated cells, at day 21. However, ESW could not evoke such an alternation in the cells pre-transfected with FAK siRNA (Fig. 2d). Quantitative data also shows that a significant inhibition of extracellular calcium deposition in the group pre-treated with *FAK* siRNA, as compared to the response to ESW in the control group without siRNA treatment (*P < 0.01*, Fig. 2d). Furthermore, the phosphorylated expression of ERK1/2 (p-ERK1/2) was intensively elevated, followed by the activation of FAK, in 4 hours post-ESW treatment, as compared to the control group, while the ERK1/2 signaling was inhibited when FAK was downregulated even though giving ESW treatment (Fig. 2e,f). The semi-quantitative results showed that there was 3-fold, 4-fold, 3-fold higher expression of p-FAK in BMSCs, TDSCs, ADSCs from OIM plus ESW group, as compared to OIM group, respectively (Fig. 2f). However, p-FAK expression was significantly lower in cells pre-treated with *FAK* siRNA before ESW treatment than that without *FAK* siRNA pre-treatment (Fig. 2f). Similar alternation in the activated form of ERK1/2 was also observed (Fig. 2f). In addition, both the expression of activated form of RUNX2 (p-RUNX2) and total RUNX2 were blocked when the cells were pre-treated with *FAK* siRNA (Fig. 2g,h). Taken together, these comprehensive data indicate that the enhancement of osteogenesis induced by ESW is mediated by the activation of FAK signaling pathway.

### miR-138 is significantly decreased post-ESW treatment

**Figure 3:**
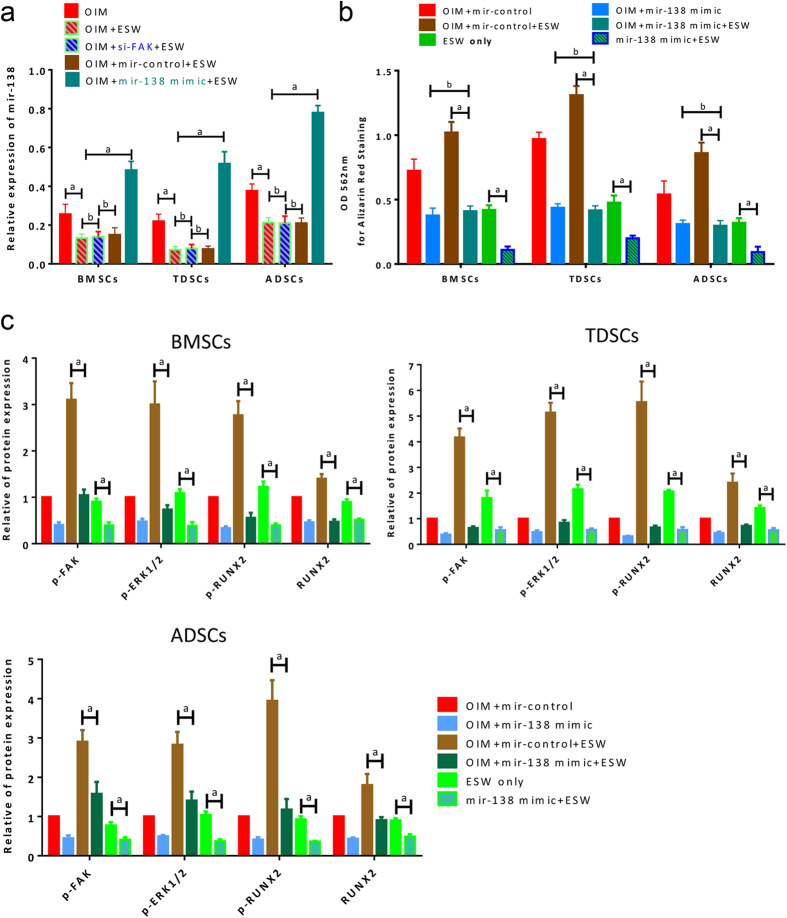
miR-138 is the upstream regulator of FAK signaling in the promotion of osteogenic differentiation induced by ESW. (**a**) Quantitative real-time PCR results show the expression of miR-138 in specified conditions. The miR-138 expression was not affected by the pre-treatment of *FAK* siRNA. (**b**) Overexpression of miR-138 dramatically abrogated the benefit effect, i.e. osteogenic promotion, from ESW, as indicated by Alizarin red staining. (**c**) The ESW induced FAK-ERK1/2-RUNX2 activation was significantly inhibited by miR-138 overexpression as shown in the western blotting results. The expression of OIM + miR-control was normalized as 1. All figure panels: representative data from at least three independent experiments using biological replicates (n = 4). Error bars, mean ± S.D. *a, P < 0.01*; *b, P > 0.05* as compared to the indicated group, using ANOVA with Bonferroni *post hoc* test.

As *FAK* gene was previously identified as a direct target for miR-138 during the osteogenic differentiation of hBMSCs^29^, we next evaluated whether miR-138 is also involved in the promotion of osteogenesis induced by ESW. As shown in Fig. 3a, miR-138 was significantly decreased when suffering from ESW treatment plus OIM as compared to OIM only group (*P < 0.01*). Similar trend was obtained in TDSCs and ADSCs (Fig. 3a). Of note, *FAK* siRNA pre-treatment did not affect the miR-138 expression (*P > 0.05*, Fig. 3a), indicating that FAK is likely the downstream target, rather than the upstream element of miR-138.

### Overexpression of miR-138 abrogates the ESW-induced osteogenesis

miR-138 mimics were successfully transfected into the cells (Fig. 3a). Results from Alizarin red staining show that transfection of miR-138 mimics significantly decreased the capability of osteogenic differentiation induced by ESW (*P < 0.01*, Fig. 3b). In addition, quantitative data from western blotting showed that the activation of FAK, ERK1/2 and RUNX2 were significantly inhibited by transfection of miR-138 mimics (Fig. 3c). These results, at least, indicate that miR-138 expression is inversely correlated with FAK during the ESW induced osteogenesis of MSCs (BMSCs, TDSCs, and ADSCs).

### The activation of ERK1/2 is FAK-dependent in the ESW-induced osteogenesis

**Figure 4:**
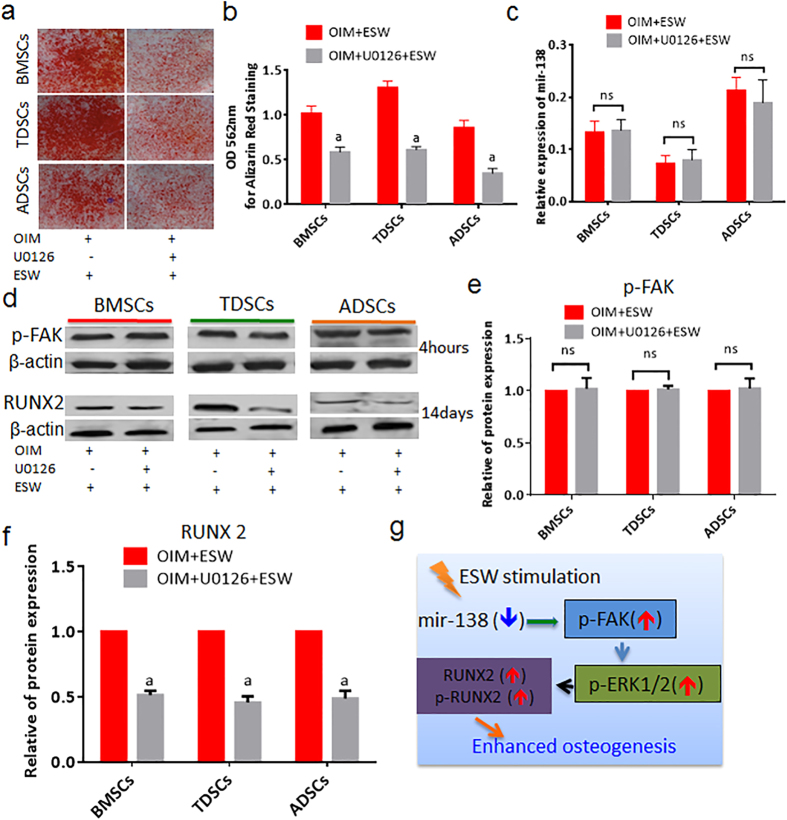
ERK1/2 is the downstream element of FAK signaling and mediates FAK induced activation and expression of RUNX2 in the response to ESW. (**a**) Alizarin red staining images shows a consistent scenario in three types of MSCs that the ESW induced osteogenesis was significantly inhibited by U0126 (20 μM), a typical ERK1/2 inhibitor. (**b**) Quantitative data from (**a**). (**c**) The expression of miR*-*138 was not affected by the pre-treatment of U0126 as measured by real-time PCR. (**d**) The expression levels of p-FAK (4 hours post-ESW treatment) and RUNX2 (14 days post-ESW treatment) in the absence or presence of U0126 were analyzed by western blotting. (**e**) Quantitative data for p-FAK expression from (**d**). (**f**) Quantitative data for RUNX2 expression from (**d**). (**g**) A scheme shows the miR-138-FAK-ERK1/2-RUNX2 pathway in the promotion of osteogenesis induced by ESW. Error bars, mean ± S.D. *a, P < 0.01* as compared to OIM plus ESW group; *ns*, not statistically significant, using Student’s *t* test. All figure panels: representative data from at least three independent experiments using biological replicates (n = 4).

To investigate the role of ERK1/2 signaling and its relationship with FAK during the ESW-induced osteogenic differentiation, U0126 (a specific ERK1/2 inhibitor) was used to pre-treat the cells those were being subjected to OIM with or without ESW treatment. It is interesting that U0126 inhibited the osteogenic differentiation of MSCs (Fig. 4a,b), suggesting that ERK1/2 activation is one of the key effector responsible for ESW treatment. However, U0126 did not affect the alternation of miR-138 and p-FAK initiated by ESW (Fig. 4c–e). These results indicate that ERK1/2 is likely the downstream element of p-FAK in ESW-induced osteogenesis.

### RUNX2 is a downstream effector of ERK1/2 during ESW-induced osteogenesis

As shown in Fig. 4d,f, the elevated expression of RUNX2 induced by ESW was significantly blocked by the pre-treatment of U0126 as compared to ESW only group, at day 14 during osteogenic induction. These results further imply that the ESW treated cells are undergoing differentiation into osteoblastic lineage, consistent with enhanced calcium deposition and ALP activity. Therefore, we can summarize a common but previously unreported molecular mechanism on how ESW stimulates the osteogenic differentiation of MSCs as Fig. 4g.

### ESW efficiently promotes the generation of new bone *in vivo*

**Figure 5:**
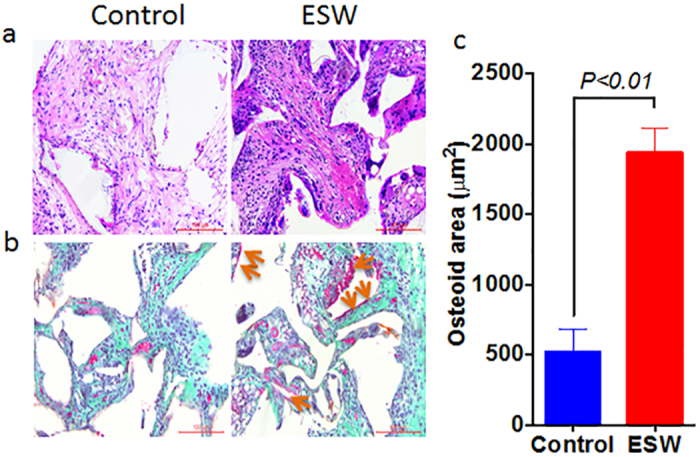
New bone formation is increased in PLGA scaffold seeded with ESW-treated TDSCs. (**a**) Routine H&E staining shows that the tissues generated inside the pores of the scaffold became more solid, as compared to those seeded with non-ESW-treated cells. (**b**) Representative trichrome masson goldner stainings shows that significantly more osteoid (purple to red) were produced post-ESW treatment. (**c**) Corresponding quantitative data shows that significant difference in the total area of osteoid between these two groups. *P* value was calculated by Student’s *t* test. Error bars, mean ± S.D. (n = 5); scale bar, 100 μm. Yellow arrows point to the osteoid.

Four weeks post the scaffolds seeded with TDSCs were implanted subcutaneously, we observed that more solid tissues formed in the ESW treated group, while there was just some unconsolidated fibrous like tissues in the control group (Fig. 5a). As indicated by Trichrome Masson Goldner staining and compared to the cells treated without ESW, ESW-treated TDSCs produced significantly more osteoid in the surface of the newly formed tissue (Fig. 5b,c).

**Figure 6:**
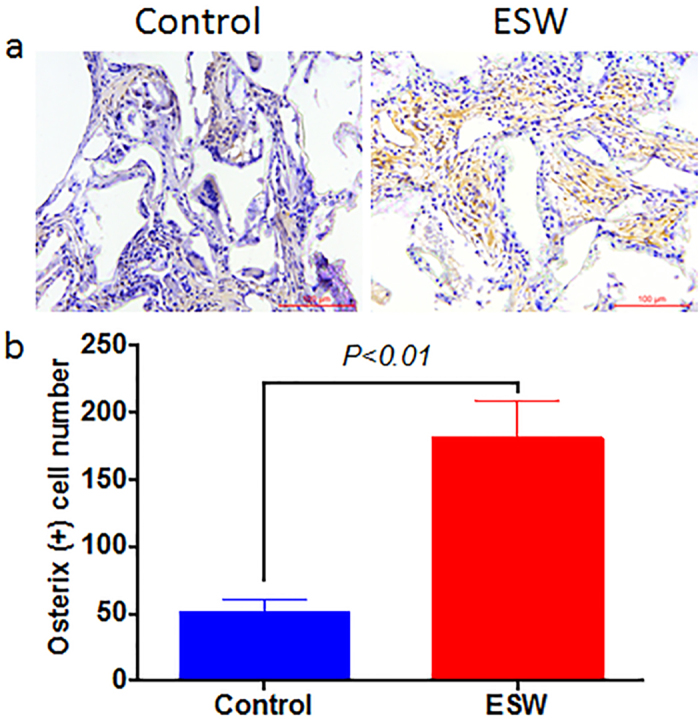
ESW treatment promotes the expression of osterix *in vivo* (**a**). (**b**) Corresponding quantitative data shows that significantly more osterix (+) cells in ESW group. *P* value was calculated by Student’s *t* test. Error bars, mean ± S.D. (n = 5); scale bar, 100 μm.

In addition, we also confirmed that there were significantly more osterix (+) cells in the newly generated tissue in ESW group, indicating higher proportion of the transplanted TDSCs were under-differentiating towards osteoblastic lineage after *in vivo* implantation, as compared to control group (*P < 0.01*, Fig. 6a,b).

## Discussion

As a unique noninvasive physical modality, ESW has attracted particular attention in the field of *in vivo* tissue regeneration, including bone^10^,^30^, tendon^31^, tendon-bone insertion^32^, and even cardiovascular system^33^. It has been suggested that ESW may exert its benefit effects via affecting the cellular behaviors, including proliferation^34^, differentiation^13^,^35^, adhesion and migration^15^,^34^. For bone tissue regeneration, it is commonly more benefit if more stem cells differentiate into bone-forming osteoblasts after ESW treatment. In fact, we and other groups have previously confirmed that medium intensity ESW (0.08–0.28 mJ/mm^2^, ref. 32) could promote the osteogenic differentiation of human BMSCs *in vitro*^9^,^13^,^36^. However, the regulatory mechanisms for these improvements have not been well addressed in these studies. In addition, based on literature review, the effect of ESW on the osteogenic differentiation of other types of MSCs, i.e. ADSCs and TDSCs, is rather limited, though emerging evidence suggest that they may bear tremendous clinical potential^16^,^17^,^19^,^21^,^27^,^37^,^38^. Recently, Schuh C. and colleagues reported that repetitive ESW treatment could enhance the stemness and preserve the multipotency of hADSCs as both the osteogenic and adipogenic differentiation of the cells were significantly increased under specified inductive culture conditions^39^. However, of note, the results from BMSCs and ADSCs are not comparable due to different devices, different energy densities, and different treatment protocols were used. It is highly desirable to investigate the unsolved, but highly significant question on whether ESW could stimulate osteogenic differentiation of different types of MSCs under the same experimental conditions (ESW generator/intensity, cell density, outcome measurements, testing parameters) and whether there is a unique signaling pathway shared by the tested MSCs via which ESW exhibits its benefit effects.

In the present report, for the first time, we reveal a common underlying mechanism that inhibition of miR-138 by ESW (0.16 mJ/mm^2^) leads to significantly activate the FAK signaling with increased phosphorylation of FAK at tyr397 site (Fig. 4g). Afterwards, ERK1/2 signaling pathway is triggered by FAK activation. Furthermore, protein levels of both the phosphorylated and total RUNX-2 (a known key transcriptional factor for osteogenesis) are dramatically increased (Fig. 4g). Consequently, ESW significantly promotes the osteogenic differentiation in the tested types of MSCs, including BMSCs, TDSCs and ADSCs.

Therefore, in our study, TDSCs and ADSCs are proven as alternative cell sources to BMSCs for bone tissue regeneration, which has key therapeutic implications. However, by paralleled comparing the response of BMSCs, TDSCs and ADSCs to ESW treatment, it is confirmed that their sensitivities are not identical as the following sequence: TDSCs > BMSCs > ADSCs. We have to bear in mind that the highly sensitivity of TDSCs to ESW treatment (0.16 mJ/mm^2^, 500 impulses) may not always that beneficial as it may induce adverse effect, i.e. calcification inside tendon tissue, if ESW is used as the treatment for tendinopathy *in vivo*. On the other hand, this finding may also provide a scientific foundation for explaining why low dose ESW (<0.08 mJ/mm^2^) is usually chosen to treat the diseased tendons^32^,^40^.

More importantly, the indispensable mechanism initiated by ESW, which goes from the inhibition of miR-138 to the elevation of RUNX2, is revealed step by step. It has been reported previously that the ectopic expression of miR-138 significantly improved the efficiency of iPS cell generation via Oct4, Sox2, and Klf4, with or without c-Myc, without sacrificing the pluripotent characteristics of the generated iPS cells^41^. Especially, miR-138 is also suggested to be a negative regulator of the BMSCs osteogenic differentiation^29^,^42^, and antimiR-138 that acts to inhibit the endogenous miR-138 level is demonstrated to enhance the *in vivo* bone formation^29^. More recently, on the top of this theory, strategy for non-viral oligonucleotide antimiR-138 delivery has been developed to meet the purpose of enhancing the osteogenic regeneration of mesenchymal stem cell sheets^43^. After confirming the central role of FAK in ESW induced osteogenesis, the upstream and downstream factors around FAK captured our attention. Here, our data on miR-138-FAK interaction is supported by gain of function of miR-138 would actually abolish the benefit effect induced by ESW via inactivating the FAK signaling, ERK1/2 and RUNX2. Therefore, for the first time, we find an alternative physical intervention, i.e. ESW, can interrupt the expression level of miR-138. In fact, our finding is consistent with that from another study, in which *FAK* is identified as the direct target for miR-138 during osteogenic differentiation of hBMSCs^29^. Regarding the downstream effectors of FAK in our study, ERK1/2 is proven as the main signaling activated by FAK, followed by RUNX2, as the osteogenic stimulation and elevation of RUNX2 induced by ESW was markedly abrogated in U0126 pretreated MSCs, without miR-138 and FAK activation affected. Likewise, we have previously reported that ERK1/2 was activated during osteogenesis^44^. Coincidentally, Ge C. and colleagues have also found that RUNX2 phosphorylation is predominantly mediated by ERK1/2, rather than p38 MAP kinase (MAPK) pathway, as ERK1/2 preferentially bound to a consensus MAPK binding site on RUNX2 that was essential for the activity of either kinase^45^.

In conclusion, ESW (0.16 mJ/mm^2^, 500 impulses) treatment can stimulate the osteogenic differentiation of human MSCs by regulating molecules in a multitude manner, whereas without the need for genetic manipulations, making this approach directly compatible with clinical applications in regenerative medicine.

## Methods

### Human rights

The Human Research Ethics Committee of Shantou University Medical College approved all relevant experiments in this study. And the work was carried out in accordance with The Code of Ethics of the World Medical Association (Declaration of Helsinki). Informed consent was obtained for experimentation with human subjects.

### Reagents and antibodies

Low/high glucose Dulbecco’s minimal essential medium (LG-DMEM, HG-DMEM), α-MEM and fetal bovine serum (FBS) were purchased from Hyclone. Collagenase type I (Cat# No.: C0130) was purchased from Sigma-Aldrich. Trypsin, antibiotics, RNA/protein extraction kits, Platinum^®^ SYBR® Green qPCR SuperMix-UDG, specific primers for human miR-138 and internal control U6, Opti-MEM^®^ I reduced serum medium, and Lipofectamine^TM^ RNAiMax were purchased from Life technologies. miScript SYBR Green PCR kit was from Qiagen (Valencia, CA). The *FAK* small interfering RNA (siRNA), siRNA control, miR-138 mimics and mimics control were synthesized according to designed sequences by GenePharma (Shanghai, China). ERK kinase inhibitor (U0126, Cat# No.: V1121) was purchased from Promega (Madison, WI). The PCR primers for *osteocalcin* and *RUNX2* were synthesized by Sangon (Shanghai, China). The PrimeScript^TM^ RT reagent kit with gDNA eraser (Cat# No.: RR047A) and easy dilution buffer (Cat# No.: 9160) were from Takara (Dalian, China). For flow cytometry, FITC-conjugated anti-human CD34 (Cat# No.: 11034942) and CD73 (Cat# No.: 11073942), Cy5.5-conjugated anti-human CD45 (Cat# No.: 45045942), and their relative conjugated IgGs (isotype controls) were purchased from E-Biosciences. PE-conjugated anti-human CD90 (Cat# No.: 328110), CD105 (Cat# No.: 323206) and relative isotype controls were from Biolegends. For western blotting, monoclonal antibodies against FAK, phospho-FAK^Tyr-397^, phospho-ERK1/2, RUNX2, phospho-RUNX2, β-actin and horseradish peroxidase-conjugated secondary antibody were purchased from Cell Signaling Technology. The SuperSignal western blotting detection kit was obtained from Pierce. The mentioned antibody for RUNX2 was also used for immunofluorescence assays, after reacting with Cy5-conjugated secondary antibody. Other chemicals and reagents were of molecular biology grade and were purchased from local commercial stores.

### The isolation and identification of human MSCs

The bone marrow, tendon and adipose tissues were obtained from surgical specimens immediately after resection from three young patients (male, average age: 26.5 years old) undergoing lower extremity amputation caused by server work-related trauma in the our institutions. About 1 g tendon tissue from the mid-substance of patellar tendon and 1 g white adipose tissue from the popliteal fossa were collected from each individual. Bone marrow (about 5 ml) was also collected. The tissues were stored in ice during the transportation to our lab. The duration was shorter than 30 mins. The well-established protocols for isolation of primary BMSCs, TDSCs and ADSCs were adopted in our study^19^,^20^,^29^,^37^. For BMSCs isolation, the collected bone marrow was mixed with 25 ml alpha-MEM containing 10% fetal bovine serum (FBS; Hyclone, Australia) and antibiotics (100 U/ml of penicillin and 100 μg/ml of streptomycin), and plated equally into three 10 cm culture dishes (Corning, USA) to culture for 5 days. On the fifth day after plating, the non-adherent cells were removed by washing with sterile PBS for three times. Then fresh alpha-MEM complete medium was added for further culture until the cells reached 80% confluence. For the isolation of ADSCs, 200 mg/ml adipose tissue was dissociated for 5–10 min in DMEM containing antibiotics, 2 mg/ml collagenase, and 20 mg/ml bovine serum-albumin. The crude stromal-vascular-fraction (SVF) was separated from the adipocyte fraction by low speed centrifugation (200 *g*, 10 min). The adipocyte fraction was discarded and cells from the pelleted SVF were seeded onto uncoated tissue culture plates (Corning, USA) at 1,000–3,500 cells/cm^2^ in LG-DMEM supplemented with 10% FBS and antibiotics as described before. Slow-adherent cells, termed CS cells, were removed after seeding onto the culture plate for 12 hours. The adherent cells in the culture plate were fast-adherent cells (CA cells) and used for further experiments. For the isolation of TDSCs, the tendon tissue was cut into small pieces (1 cm^3^) and subjected with 0.1% collagenase type I digestion in LG-DMEM containing 10% FBS for overnight. The remaining tissues were removed and dissociated cells were collected and cultured in complete culture medium. The isolated MSCs were cultured at 37 °C, 5% CO_2_. When sub-cultured the MSCs by digesting the adherent cells using 0.05% trypsin (Life technologies) for 1 min and stopping digestion using complete medium containing 10% FBS, the cells were collected and seeded at a density of 4,000 cells/cm^2^ onto 75 cm^2^ flasks (NUNC, Denmark). Medium was changed every 2 days. Flow cytometry was used to determine the expression levels of known surface markers (positive markers: CD73, CD90, CD105; negative markers: CD34, CD45) in these three types of MSCs (all at Passage 3), according to our published protocol^15^. After identified as MSCs with high purity, the cells were used in subsequent experiments. All the cells used in this study were at passage 3–5.

### ESW treatment

Based on our and other previous studies, the optimal intensity for ESW is set as with an energy flux of 0.16 mJ/mm^2^, for 500 impulses^9^,^15^. Before ESW treatment, the stem cells (normal, siRNA pre-treated, miR-138 overexpressed, U0126 pre-treated) were washed and re-suspended with complete medium. Cells were suspended with 2 ml culture medium in 15-ml sterile polystyrene tubes (Corning, USA) at a concentration of 5 × 10^5^/ml. ESW was delivered onto the firmly attached surface of the tube containing cells using a shockwave generator (Dornier MedTech Epos, Wessling, Germany) according to our established protocol (Fig. 1c)^15^. The duration of each ESW treatment lasted 10 minutes. After ESW treatment, the cells were placed onto plastic dishes or culture plates for different times as required. Respective MSCs without ESW treatment were run as controls.

### Osteogenic induction and assays

The MSCs were plated at 4.0 × 10^3^ cells/cm^2^ in 12-well plates and cultured in basal complete culture medium until the cells reached confluence. They were then incubated in basal complete medium (HG-DMEM with 10% FBS) or osteogenic medium (complete culture medium supplemented with 10^−8^ M dexamethasone, 50 μM L-ascorbic acid, 20 mM β-glycerophosphate). Three sets of samples were prepared for the well-established quantitative RT-PCR (qRT-PCR), Alizarin red staining, measurement of ALP activity^20^. At day 14, the mRNA expression of osteogenic markers (*Osteocalcin* and *RUNX2*) was assessed using qRT-PCR as described below. At day 21, the calcium nodule formation in BMSCs, TDSCs and ADSCs was assessed using Alizarin red S staining assay. The stained plates were firstly dried and scanned using a scanner (HP). Then, to quantitate the amount of Alizarin red bound to the mineralized nodules, cells were rinsed with water, and extracted with 10% (w/v) cetylpyridinium chloride (CPC) in 10 mM sodium phosphate, pH 7.0 for 15 minutes at room temperature. The dye concentration in the extracts was determined at OD 562 nm. ALP activity, another important parameter for determining the osteogenesis, was also performed at day 21. In brief, cells of each well were washed and lysed with lysis buffer (500 μl) with protease inhibitor cocktail (BD). The supernatant was assayed for ALP activity with an ALP assay kit (BioSystems, Barcelona, Spain). Production of *p-* nitrophenol was measured at OD 405 nm.

Moreover, immunofluorescence assay was used to detect if the expression of RUNX2 in the cells treated by ESW is different with those from control during osteogenic induction. In brief, MSCs from different groups were plated at 1.0 × 10^3^ cells/cm^2^ onto the sterilized coverslip in 6-well plates and cultured in basal complete culture medium. After the cells fully spread, we changed the medium into osteogenic induction medium. The medium was refreshed every two days. At day 14, the cells were washed and fixed in 4% paraformaldehyde in PBS (pH 7.4) for 15 minutes. Antigen was retrieved by heated in the citrate butter (pH 6.0). Afterwards, nonspecific binding was blocked with 10% serum from the species that the secondary antibody was raised in PBS with 1% bovine serum albumin (blocking buffer) for 30 minutes at room temperature. Primary antibody to RUNX2 in the same blocking buffer (v/v, 1:100) were applied to the sections overnight at 4 °C. After washing, Cy5-conjugated secondary antibody (v/v, 1:100) in blocking buffer was added to the sections for 1 h at room temperature in the dark. The coverslips were then counterstained with the nuclear stain 4′, 6- diamidino-2-phenylindole in ProLong Gold Antifade Reagent (Life Technology, Carlsbad, CA). The coverslips were mounted and examined with a fluorescent microscope equipped with a UV laser (ZEISS AxioPlan 2; Carl Zeiss MicroImaging LLC, Jena, Germany) at different emission and excitation wavelengths.

### Quantitative real-time PCR

RNA isolation and reverse transcription were performed as previously described^20^. Specific primers were used: *Osteocalcin* (forward, 5′-ACACTCCTCGCCCTATTG-3′, reverse, 5′-GATGTGGTCAGCCAACTC-3′), *RUNX2* (forward, 5′-GGTTAATCTCCGCAGGTCACT-3′; reverse, 5′-CACTGTGCTGAAGAGGCTGTT-3′), *β-actin* (forward, 5′-GGCACCCAGCACAATGAAG-3′; reverse, 5′-TGC GGTGGACGATGGAGG-3′). Quantitative RT-PCR was performed with the double-stranded DNA-binding dye SYBR Green on the ABI 7900 Real-Time PCR System (Applied BioSystems, Inc.), and relative gene expression was analyzed as indicated by the manufacturer.

The expression of miR-138 was detected by qRT-PCR using miScript SYBR Green PCR Kit according to the manufacturer’s protocol from Qiagen. U6 expression was used as internal control. The primers for microRNAs were from Life technologies.

### Western blot analyses

Total protein was isolated from cells cultured in dishes (Φ = 10 cm) by using RIPA lysis buffer as established in our lab^15^,^44^. Equal amount proteins (20 μg) from different samples were separated by 12% SDS/PAGE and electrotransferred into PVDF membranes; membranes were incubated with primary antibodies against FAK (v/v, 1:1000), p-FAK (v/v, 1:1000), p-ERK1/2 (v/v, 1:1000), RUNX2 (v/v, 1:1000), p-RUNX2 (v/v, 1:1000), and β-actin (v/v, 1:1000) overnight at 4 °C, respectively. Membranes were incubated with HRP conjugated secondary antibody (v/v, 1:10000) for 1 hour at room temperature, and protein bands were visualized with ECL chemiluminescence detection system. Densitometric analysis involved the use of Quantity One software (version 4.5.2; Bio-Rad). The expression of β-actin was shown as a control for equal protein loading.

### Signaling pathway analyses

To test our hypothesis and investigate the molecular mechanism thoroughly, the following oligonucleotides were used in the current study: miR-138 mimics^29^, 5′-AGCUGGUGUUGUGAAUCAGGCCG-3′ (sense); control mimic, 5′-CUCCGAACGUGUCACGU-3′ (sense); *FAK* siRNA^46^, 5′-GGAGUGGAAAUAUGAAUUGTT-3′ (sense); 5′-CAAUUCAUAUUUCCACUCCTC-3′ (anti-sense). In this study, we optimized the experimental conditions for transfection as following: the same initial cell seeding density (at passage 3), 4 × 10^3^ cell/cm^2^; cultured in medium without antibiotics and serum; concentration, 50 nM for both; required reaction time, miR-138 mimics (24 hours)/*FAK* siRNA (48 hours). The conditional medium was a mixture of specified oligonucleotide, Opti-MEM^®^ I reduced serum medium, and Lipofectamine^TM^ RNAiMax as mentioned^15^. At these optimal transfection conditions, the cell proliferation was not affected (data not shown).

ERK1/2 kinase inhibitor, named U0126, was also used as a critical tool to analysis the downstream effectors. MSCs were treated with 20 μM U0126 (did not affect cell proliferation) for 60 min prior to ESW treatment.

Cells were washed and re-suspended before they were subjected to the ESW treatment as described above. After ESW treatment, cells were incubated for a certain additional period of time, under different conditions.

### Measurement of bone formation in nude mice after subcutaneously implantation

We sought to demonstrate the osteoinductive effect of ESW *in vivo*. The Poly (_D,L_-lactide-*co*-glycolide) (PLGA) (L/G ratio 50:50, MW 40,000–75,000) with 80% porosity (average diameter of ~250 μm) were produced by Shanghai Biotech. Ltd. Institutional Animal Care and Use Committee approval was obtained from Shantou University Medical College before beginning all animal studies. Total 10 nude mice aged 12 weeks were used. Human TDSCs treated with or without ESW (5 × 10^5^) were seeded onto the sterile PLGA scaffolds with equal size of 5 × 5 × 5 mm^3^ and incubated in complete culture medium for another 1 hour before implantation. After the nude mouse was anesthetized using intraperitoneal injection (3.5 mg/20 g body weight) of Ketamine, an incision was made on the dorsum and a subcutaneous pocket was created. Next, the PLGA scaffolds with ESW-treated (n = 5) or non-ESW-treated (n = 5) TDSCs were transplanted respectively. The wound was closed with 5-0 silk sutures. At week 4 post surgery, the implanted scaffolds were harvested and fixed with 4% buffered formalin and then embedded into paraffin wax before sectioning. Sections with 5-μm thickness were prepared and routinely stained with hematoxylin-eosin (H&E). Trichrome masson goldner staining was also performed for static histomorphometric measurement. A semi-automatic digitizing image analysis system (OsteoMetrics, Atlanta, GA, USA) was used to measure the area of osteoid. The expression of transcriptional factor, osterix, was determined by immunohistochemical staining. Specific antibody against osterix (Cat.# ab94744; Abcam, Cambridge, Massachusetts, USA; dilution: 1:200) and goat anti-rabbit IgG horseradish peroxidase (HRP)-conjugated secondary antibody (dilution: 1:200) were used, and the binding was visualized using DAB Quanto kit according to the manufacturer’s protocol (Thermal Scientific, California, USA).

### Statistical analyses

The data presented are the means ± S.D. (standard deviation) of at least three experiments performed in triplicate. All data demonstrated a normal distribution and similar variation between groups. Student’s unpaired *t* test was used to compare difference between two groups, while one-way analysis of variance (ANOVA) with Bonferroni *post hoc* test was used for multiple comparisons in GraphPad Prism 6.0 (GraphPad Software, Inc., USA). Statistical significance was determined as *P < *0.05.

## Additional Information

**How to cite this article**: Hu, J. *et al.* Focal Adhesion Kinase Signaling Mediated the Enhancement of Osteogenesis of Human Mesenchymal Stem Cells Induced by Extracorporeal Shockwave. *Sci. Rep.* **6**, 20875; doi: 10.1038/srep20875 (2016).

## Supplementary Material

Supplementary Information
