# Supplementary material for: Focal Adhesion Kinase Signaling Mediated the Enhancement of Osteogenesis of Human Mesenchymal Stem Cells Induced by Extracorporeal Shockwave
Source: Sci Rep. 2016 Feb 11;6:20875. doi: 10.1038/srep20875 (PMC4750003; doi:10.1038/srep20875)
Supplement: Supplementary Information [file srep20875-s1.pdf]

Supplementary Information

Focal Adhesion Kinase Signaling Mediated the Enhancement of Osteogenesis of Human Mesenchymal Stem Cells Induced by Extracorporeal Shockwave

Jun Hu[1, *], Haojie Liao[1], Zebin Ma[1], Hongjiang Chen[1], Zhonglian Huang[1], Yuantao Zhang[1], Menglei Yu[3], Youbin Chen[1, a], Jiankun Xu[1, 2, a]

Supplementary figure 1

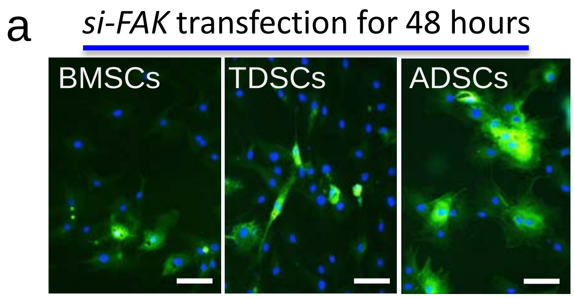

Figure S1. Fluorescein isothiocyanate (FITC) signal was detectable in the *si-FAK* transduced cells, indicating that *si-FAK* was successfully transported into the cultured cells. Scale bars, 50 μm.
